# Modulatory effects of *Tabebuia impetiginosa* (Lamiales, Bignoniaceae) on doxorubicin-induced somatic mutation and recombination in *Drosophila melanogaster*

**DOI:** 10.1590/S1415-47572009005000042

**Published:** 2009-05-01

**Authors:** Neila C. de Sousa, Alexandre A. A. de Rezende, Regildo M. G. da Silva, Zaira R. Guterres, Ulrich Graf, Warwick E. Kerr, Mário A. Spanó

**Affiliations:** 1 Universidade Federal de Goiás, Campus Catalão, Catalão, GOBrazil; 2 Instituto de Genética e Bioquímica, Universidade Federal de Uberlândia, Uberlândia, MGBrazil; 3 Physiology and Animal Husbandry, Institute of Animal Sciences, ETH ZurichSwitzerland

**Keywords:** genotoxicity, synergistic effect, somatic mutation and recombination test - SMART, toxicity, wing spot test

## Abstract

The wing Somatic Mutation and Recombination Test (SMART) in *D. melanogaster* was used to study genotoxicity of the medicinal plant *Tabebuia impetiginosa*. Lapachol (naphthoquinone) and β-lapachone (quinone) are the two main chemical constituents of *T. impetiginosa*. These compounds have several biological properties. They induce apoptosis by generating oxygen-reactive species, thereby inhibiting topoisomerases (I and II) or inducing other enzymes dependent on NAD(P)H:quinone oxidoreductase 1, thus affecting cell cycle checkpoints. The SMART was used in the standard (ST) version, which has normal levels of cytochrome P450 (CYP) enzymes, to check the direct action of this compound, and in the high bioactivation (HB) version, which has a high constitutive level of CYP enzymes, to check for indirect action in three different *T. impetiginosa* concentrations (10%, 20% or 40% w/w). It was observed that *T. impetiginosa* alone did not modify the spontaneous frequencies of mutant spots in either cross. The negative results observed prompted us to study this phytotherapeuticum in association with the reference mutagen doxorubicin (DXR). In co-treated series, *T. impetiginosa* was toxic in both crosses at higher concentration, whereas in the HB cross, it induced a considerable potentiating effect (from ~24.0 to ~95.0%) on DXR genotoxity. Therefore, further research is needed to determine the possible risks associated with the exposure of living organisms to this complex mixture.

## Introduction

*Tabebuia impetiginosa* (Lamiales, Bignoniaceae), popularly known as Ipê, pau d'arco, pink trumpet tree, taheebo and lapacho rosado, is a medicinal plant, native to tropical rain forests and the ‘cerrado' (savannah) throughout Central and South America. *Tabebuia spp* is used in the construction of external structures, stairs and parquets (Algranti *et al.*, 2005). *Tabebuia impetiginosa* (Martius ex DC) Standley has been used in folk medicine as a diuretic and astringent, as well as for treating ulcers, syphilis, gastrointestinal problems, candidiasis, cancer, diabetes, prostatitis, constipation and allergies (Almeida, 1993; Park *et al.*, 2003).

The bark of the *Tabebuia spp* stem is a source of furanonaphthoquinones, quinines, naphthoquinones, benzoic acid, benzaldehyde derivatives, cyclopentene dialdehyde and flavonoids (Zani *et al.*, 1991; Koyama *et al.*, 2000; Park *et al.*, 2003). Further constituents from the bark of *T. impetiginosa* are iridoid glycosides, lignan glycosides, isocoumarin glycosides, phenylethanoid glycosides and phenolic glycosides (Warashina *et al.*, 2004; 2005; 2006). Lapachol [2-hydroxy-3-(3-methyl-2-butenyl)-1,4-naphtalene-dione], naphthoquinone and its derivative β-Lapachone (2,2-dimethyl-3,4-dihydro-2,4-benzo[h]chromene-5,6-dione), which possess biologically active properties, can be isolated from *T. impetiginosa* (Park *et al.*, 2003). The inner bark extract of this plant potently inhibited cell proliferation and DNA synthesis (Son *et al.*, 2006). The stereo-selective synthesis of biologically active naphthoquinones from *Tabebuia avellanedae*, as described by Yamashita *et al.* (2007), displayed potent cytotoxicity against several human tumor cell lines, whereas it showed lower cytotoxicity against certain normal human cell lines when compared with that of mitomycin. A synthetic version of the natural product β-lapachone has been isolated from *T. impetiginosa*, and has also demonstrated promising anticancer activity (Savage *et al.*, 2008).

The *T. impetiginosa* bark compounds lapachol and β-lapachone are reportedly antipsoriatic, antifungal, antimicrobial, antioxidant, antiviral, anti-inflammatory, antiulcerogenic, anticarcinogenic, antibacterial and antimalarial, besides possessing antitrypanosomal activity and acting as a chemoprophylactic against infection by *Schistosoma mansoni* cercariae (Anesini and Perez, 1993; Müller *et al.*, 1999; Fonseca *et al.*, 2003; Park *et al.*, 2003*,* 2005, 2006; Menna-Barreto *et al.*, 2005). An overview of Lapachol is presented by Hussain *et al.* (2007).

The anthracycline antibiotic doxorubicin is a topoisomerase II inhibitor (Swift *et al.*, 2008) and a generator of oxygen free radicals (Doroshow, 1983). Previous studies have shown that DXR induces preferentially homologous recombination compared with mutational events in somatic cells of *D. melanogaster* (Lehmann *et al.*, 2003; Fragiorge *et al.*, 2007; Pereira *et al.*, 2008; Silva *et al.*, 2008; Valadares *et al.*, 2008).

The wing spot test in *Drosophila melanogaster* (Somatic Mutation and Recombination Test - SMART) is a versatile, efficient and inexpensive short-term *in vivo* genotoxicity assay for the detection of genotoxicity induced by single pure compounds and complex mixtures. For this reason, it is also ideally suited for anti-genotoxicity studies as well as for investigations into the modulation of genotoxicity (Graf *et al.*, 1998). It was developed to detect the loss of heterozygosity of the marker genes expressed phenotypically in the trichomes of the fly's wings. It provides rapid information on the ability of genotoxic agents to induce (or of antigenotoxic agents to inhibit) point mutations, chromosome breaks or losses during cell division, or the rearrangement related to mitotic recombination (Graf *et al.*, 1984; 1989; Guzmán-Rincón and Graf, 1995; Vogel *et al.*, 1999). Previous studies have shown that SMARTs are best suited for the detection of recombinogenic activity of genotoxic chemicals (Spanó *et al.*, 2001). The standard version presents basal levels of cytochrome P450 (CYP) enzymes (Graf *et al.*, 1989), whereas the high bioactivation version presents a high level of CYP (Graf and van Schaik, 1992) with the capacity to activate promutagens and procarcinogens enzymatically (Frölich and Würgler, 1990; Graf and Singer, 1992; Graf and van Schaik, 1992).

For antigenotoxicity and modulatory studies, the SMART assays offer a wide variety of flexible protocols for the application of test compounds (Graf *et al.*, 1998). Owing to these advantages, SMART has been adopted for the genotoxicity / antigenotoxicity / modulatory testing of natural products (Sousa *et al.*, 2003; Fragiorge *et al.*, 2007; Pereira *et al.*, 2008; Valadares *et al.*, 2008).

Due to the wide distribution of the genus *Tabebuia*, consisting of about 20 species of trees, its use in folk medicine and the lack of information related to genetic toxicology, it is important to evaluate: i) the genotoxicity of its leaves, stem bark, pods, and seed extracts, as these may be potentially mutagenic, clastogenic, recombinogenic, and/or carcinogenic in man; ii) its modulatory effects, which enable its use as a chemotherapeutic coadjuvant.

The aim of this study was to evaluate the genotoxic potential of a commercially available product of *T. impetiginosa* bark and stem, since this is a natural product widely used in folk medicine in Brazil. The negative results observed with *T. impetiginosa* in the somatic cells of *D. melanogaster* prompted us to study this phytotherapeuticum in association with the reference mutagen DXR.

## Material and Methods

### Chemical compounds and media

A commercial preparation of the powdered bark and trunk of *T. impetiginosa* - Ipê Roxo Max^®^ - was obtained from Saúde na Rede (Rio de Janeiro, RJ, Brazil). Doxorubicin (DXR) - Korea United Pharm Inc Co., Ltd. (Seoul, Korea) - was obtained from Meizler Comércio Internacional S.A. (Barueri, SP) and dissolved in ultrapure water in the absence of light. Ultrapure water, used as a negative control, was obtained from a MilliQ system (Millipore, Vimodrone, Milan, Italy). All solutions were always freshly prepared in ultrapure water immediately before use.

### *Drosophila* strains

Three strains were used for crossbreeding: (i) multiple wing hairs (mwh): *mwh/mwh*; (ii) flare-3 (*flr*^*3*^*/In (3LR)TM3, rip*^*p*^*sep l(3)89Aabx*^*34e*^*e Bd*^*S*^); and (iii) ORR; flare-3 (*ORR/ORR; flr*^*3*^*/In (3LR)TM3, rip*^*p*^*sep l(3)89Aabx*^*34e*^*e Bd*^*S*^). More details on the genetic markers are given by Lindsley and Zimm (1992).

### Crossbreedings for the SMART assays

Two crosses were carried out: (1) the Standard (ST) cross, where flare-3 females were mated with mwh males (Graf *et al.*, 1989); and (2) the High Bioactivation (HB) cross, where ORR; flare-3 females were mated with mwh males (Graf and van Schaik, 1992). The latter cross is highly sensitive to promutagens and procarcinogens due to the increased level of CYP. The ORR; flare-3 strain has chromosomes 1 and 2 substituted in the wild DDT-resistant Oregon R (R) strain, and the gene (R) of chromosome 2 is responsible for the high constitutive level of CYP enzymes (Dapkus and Merrell, 1977; Frölich and Würgler, 1989).

Two types of individuals emerge from both ST and HB crossbreeding: marker trans-heterozygous (MH) flies (*mwh* +/+ *flr*^*3*^) and balancer-heterozygous (BH) flies (*mwh* +/+*TM3*, *Bd*^*S*^). The latter can be distinguished phenotypically by its serrated wings.

### Experimental procedure

After two days of crossbreeding, the couples were transferred to the oviposition medium (an agar-agar base (3% w/v) and a layer of fermenting live baker's yeast supplemented with sucrose) where they remained for 8 h, after which they were discarded. Third instar larvae (72 h ± 4 h) were transferred to glass vials containing different quantities (10%, 20% or 40% w/w) of powdered bark and stem of *T. impetiginosa* mixed with mashed potato flakes (Yoki Alimentos S. A. - São Bernardo do Campo, SP, Brazil), lightly ground by using a mortar and pestle, and rehydrated with 5 mL of ultrapure water (to evaluate the genotoxic effects of *T. impetiginosa*) or DXR (0.125 mg mL^-1^) (to evaluate the modulatory effects *of T. impetiginosa*). Negative (ultrapure water) and positive (DXR 0.125 mg mL^-1^) controls were included in both experiments.

The larvae were kept in the culture media at a temperature of 25 °C and relative humidity of 65% until the adult stage. Emerged adult flies were stored in 70% ethanol. The wings were mounted on glass slides and analyzed by optical microscopy with 400 x magnification, revealing single spots (mwh or flr) or twin spots (mwh and flr). For further details of this procedure, see Graf *et al.* (1984).

### Evaluation of the data and statistical analysis

The frequency of spots per fly in each series was compared with the negative control to evaluate genotoxic effects. In order to assess antigenotoxic effects, the frequency of spots per fly in each treated series was compared with the positive control. Statistical comparisons were made using the SMART computer program, which uses the chi-square test for proportions and allows for a multi-decision procedure (Frei and Würgler, 1988). For final statistical analysis of all positive outcomes, the non-parametric Mann-Whitney *U*-test with significance levels α = β = 0*.*05 was used in order to exclude false positives (Frei and Würgler, 1995). Based on clone induction frequencies per 10^5^ cells, recombinogenic activity was calculated as: mutation frequencies (*F*_M_) = frequencies clones BH flies/frequencies clones MH flies; recombination frequencies (*F*_R_) = 1- *F*_M_. Frequencies of total spots (*F*_T_) = total spots in MH flies (considering *mwh* and *flr*^*3*^ spots)/No. of flies; mutation = *F*_T_ x *F*_M_; recombination = *F*_T_ x *F*_R_ (Santos *et al.*, 1999; Sinigaglia *et al.*, 2006). Based on control-corrected spot frequencies per 10^5^ cells, the percentages of *T. impetiginosa* inhibition were calculated as: (DXR alone - *T. impetiginosa* plus DXR / DXR alone) x 100 (Abraham, 1994).

## Results

Prior to genotoxicity assessment, the commercial preparation of the powdered bark and stem of *T. impetiginosa* was submitted to a dose-range test (data not shown), which demonstrated that *T. impetiginosa* presented toxicity in larvae fed for 48 h. The non-toxic (10 and 20%) and less-toxic (40%) concentrations from all those tested were used to perform mutagenic/recombinogenic evaluation. *T. impetiginosa* (10%, 20% or 40% w/w) alone, the reference mutagen (DXR 0.125 mg mL^-1^) alone and *T. impetiginosa* (10%, 20% or 40% w/w) in association (co-treatment) with DXR 0.125 mg mL^-1^ were assayed twice in ST and HB crossbreeds. Concurrent negative and positive controls were also included. Since no statistical differences were found among the results of individual experiments, data were pooled. Tables 1  and 2 present the results observed with MH and BH flies of, respectively, the ST and HB crossbreeds. To assess the statistical significance of the frequency of mutant spots observed among flies treated with *T. impetiginosa,* the results were compared with data from the corresponding negative controls. No significant differences in the frequency of mutant spots were observed among flies treated with all the *T. impetiginosa* concentrations and the negative control in ST and HB crossbreeds MH flies.

To evaluate the statistical significance of co-treatment series, the results of the different *T. impetiginosa* concentrations in association with DXR were compared with the positive control.

In the MH flies of the ST crossbreed, the frequency of mutant spots observed between those co-treated with *T. impetiginosa* 10% or 20% and DXR showed no statistical significance, but *T. impetiginosa* 40% in association with DXR presented a weak positive diagnosis, displaying a 36.63% inhibition of DXR genotoxicity. The wings of BH flies were mounted and analyzed whenever a positive response was obtained in the MH offspring. In this case, as no significant differences in the frequency of mutant spots were observed among flies co-treated with *T. impetiginosa* 10% or 20% and DXR compared to flies treated with DXR alone, the BH flies from these treated series were not analyzed. When the BH flies co-treated with *T. impetiginosa* 40% w/w and DXR were checked, an inhibitory effect against the frequency of total spots (26.08%) was observed. Comparisons between the clone induction frequencies per 10^5^ cells observed in the MH and BH flies of the co-treated series with DXR and *T. impetiginosa* 40%, were done to quantify the mutagenic and recombinogenic potential of *T. impetiginosa.* The results showed that the genotoxicity in MH flies was mainly due to mitotic recombination (~90%) (Table 1).

In MH flies of the HB crossbreed, the frequency of mutant spots observed among those co-treated with *T. impetiginosa* 10%, 20% or 40% and DXR showed a positive increase (respectively, 94.61; 76.25 and 23.75%) in the total number of spots, when compared with DXR alone. When the BH flies treated with *T. impetiginosa* 10% w/w in association with DXR were checked, a positive increase (119.33%) in the total number of spots was noted. When comparisons between the clone induction frequencies per 10^5^ cells observed in the MH and BH flies of the co-treated series with DXR and all concentrations of *T. impetiginosa*, were done, the results showed that the enhancement of genotoxicity in MH flies was mainly due to mitotic recombination (87%-90%) (Table 2).

## Discussion

Our study shows that a commercial preparation of powdered bark and stem of *T. impetiginosa* was toxic but did not induce somatic mutation and recombination in *D. melanogaster* from ST and HB crossbreeding. This means that *T. impetiginosa* alone neither acts as a genotoxin nor exerts any antigenotoxic effects on spontaneous DNA lesions. Nevertheless, this toxicity is revealed by the diminished number of treated survivors.

DXR produced statistically significant induction of all categories of spots in both the ST and HB crossbreeds. These results are in line with those reported by Lehmann *et al.* (2003), Fragiorge *et al.* (2007), Pereira *et al.* (2008) and Valadares *et al.* (2008), who also demonstrated that DXR is a preferential inducer of homologous recombination, when compared with mutational events in *D. melanogaster* somatic cells.

As can be seen from the data in Table 1 MH flies from the ST crossbreed, no differences were observed among the frequencies of total spots induced by co-treatments with *T. impetiginosa* 10% or 20% and DXR, when compared to those from the positive control (DXR alone). However, *T. impetiginosa* 40% in association with DXR displayed a 36.63% inhibition of DXR genotoxicity. Nevertheless, this is not interpreted as an antigenotoxic or protective effect of *T. impetiginosa*, since the number of survivor flies was reduced, thereby indicating *T. impetiginosa* toxicity which could be responsible for cell death or mitotic inhibition. According to Zeiger (2006), if the test concentrations used are near the stationary or toxic level on a plate test, a slight increase in toxicity could lead to lower survival rates, with a parallel decrease in mutant colonies. Such an apparent antimutagenic effect would therefore be the result of toxicity rather than antimutagenicity. The same should be true for the results found in the present study.

Herbal/dietary constituents may be metabolized by CYP into nontoxic metabolites and excreted, but the formation of toxic metabolites is possible. In addition, the inhibition of CYPs by herbal constituents may decrease the formation of toxic metabolites and thus inhibit carcinogenesis, as CYPs play an important role in procarcinogen activation. The bioactivation of herbal constituents appears to be a critical step for toxicity induction in some herbs. The resultant reactive intermediates bind covalently to DNA and proteins, leading to organ toxicity and even carcinogenicity. On the other hand, some herbal/dietary constituents were shown to form reactive intermediates capable of irreversibly inhibiting various CYPs (Zhou *et al.*, 2004).

The results shown in Table 2 indicate the significant potentiating action of *T. impetiginosa* when administered simultaneously with DXR, and which was inversely proportional to the concentrations applied, once more indicating a dose-response correlationship with toxicity. *T. impetiginosa* 10% or 20% significantly increased DXR-induced genotoxicity, which affected all categories of spots. Nevertheless, in the case of *T. impetiginosa* 40%, synergistic activity was not so pronounced, with the significant increase in DXR genotoxicity being limited to the frequency of total spots. The magnitude of comutagenicity was considerable, leading to enhancements from 23.75 to 94.61%.

Medicinal herbs contain complex mixtures of thousands of components that can exert their action separately or synergistically (Cai *et al.*, 2004; Romero-Jiménez *et al.*, 2005). It has been well established that the formation of reactive drug metabolites is associated with drug toxicity. Similarly, data are accumulating which suggest the role of the formation of reactive metabolites/intermediates through bio-activation in herbal toxicity and carcinogenicity. It has been hypothesized that the resultant reactive metabolites following herbal bio-activation covalently bind to cellular proteins and DNA, thus leading to toxicity via multiple mechanisms such as direct cytotoxicity, oncogene activation and hypersensitivity reactions (Zhou *et al.*, 2004).

The mechanisms used by *T. impetiginosa* to interact with the genotoxicity of DXR were not analyzed directly. The results observed in the present study allow us to hypothesize that: i) *T. impetiginosa* constituents may interact with those enzyme systems catalyzing the metabolic detoxification of DXR, leading to the enhancement of DXR mutagenicity; ii) *T. impetiginosa* constituents generate superoxide radicals and stimulate microsomal oxidation via NAD(P)H. Similar results and conclusions were described by Lehmann *et al.* (2000) with tannic acid in association with mitomycin C, methylmethanesulfonate and nitrogen mustard in somatic cells of *D. melanogaster*.

Our findings demonstrated that powdered bark and stem of *T. impetiginosa* was toxic, but not genotoxic by itself, yet it possesses a considerable potentiating effect on DXR genotoxity, thereby suggesting that *T. impetiginosa* may possess anticarcinogenic potential. Therefore, further experiments, including carcinogenicity tests, are required on dose response, appropriate combinations, and potential toxicity/genotoxicity of *T. impetiginosa* associations with chemotherapeutic drugs, to determine the possible risks or protection that could be associated with the exposure of living organisms to this complex mixture.

## Figures and Tables

**Table 1:** - Summary of results obtained with the *Drosophila* Wing spot test (SMART) in the marker trans-heterozygous (MH) and balancer-heterozygous (BH) progeny of the standard (ST) cross after chronic treatment of larvae with *Tabebuia impetiginosa* (*Ti*) and doxorubicin (DXR).

Genotypes and treatment		Number of flies	Spot for fly (number of spots) statistical diagnosis^a^				Spots with mwh clone^b^ (n)	Frequency of clone formation/10^5^ cells^c^		Recombination (%)	inhibition^d^ (%)
DXR (mg mL^-1^)	*Ti* (%)		Small single spots (1-2 cells)^e^	Large single spots (> 2 cells)^e^	Twin spots	Total spots		Observed	Control corrected		
*MH*^*3*^											
0	0	55	0.44 (24)	0.04 (02)	0.04 (02)	0.51 (28)	27	1.01			
0	10	40	0.51 (28)	0.13 (07)	0.00 (00)	0.64 (35)	34	1.27	0.26		
0	20	55	0.38 (21)	0.07 (04)	0.02 (01)	0.47 (26)	26	0.97	-0.04		
0	40	54	0.57 (31)	0.04 (02)	0.02 (01)	0.63 (34)	34	1.29	0.28		
0.125	0	40	2.40 (96)+	2.33 (93)+	2.60 (104)+	7.33 (293)+	276	14.14	13.22	88.70	
0.125	10	40	2.08 (83)	2.13 (85)	3.78 (151)*	7.98(319)	306	15.68	14.75		
0.125	20	40	2.13 (85)	2.20 (88)	3.10(124)	7.43(297)	288	14.75	13.83		
0.125	40	38	1.71 (65)*	1.39 (53)*	1.79 (68)*	4.89(186)*	174	9.38	8.41	89.80	36.63
*BH*^*3*^											
0	0	40	0.15 (06)	0.00 (00)	^f^	0.15 (06)	6	0.31			
0.125	0	40	0.75 (30)+	0.08 (03)		0.83 (33)+	33	1.69	1.38		
0.125	40	40	0.40 (16)*	0.10 (4)		0.50 (20)*	20	1.02	1.02		26.08

MH flies (*mwh/flr*^*3*^) and BH flies (*mwh/TM3*) were evaluated. ^a^Statistical diagnoses according to Frei and Würgler (1995). *U*-test, two-sided, probability levels: +, p ≤ 0.05 *vs.* untreated control; *, p ≤ 0.05 *vs.* DXR only. ^b^Considering *mwh* clones from *mwh* single and twin spots. ^c^Frequency of clone formation: clones/flies/48,800 cells (without size correction). ^d^Calculated as [DXR alone - (DXR + *Ti*) / DXR alone] X 100, according to Abraham (1994). ^e^Including rare *flr*^*3*^ single spots. ^f^Only mwh single spots can be observed in BH individuals.

**Table 2:** - Summary of results obtained with the *Drosophila* wing spot test (SMART) in the marker-trans-heterozygous (MH) and balancer-heterozygous (BH) progeny of the high bioactivation (HB) cross after chronic treatment of larvae with *Tabebuiaimpetiginosa* (*Ti*) and doxorubicin (DXR).

Genotypes and treatment		Number of flies	Spot for fly (number of spots) statistical diagnosis^a^				Spots with mwh clone^b^ (n)	Frequency of clone formation/10^5^ cells^c^		Recombination (%)	Induction^d^ (%)
DXR (mg mL^-1^)	*Ti* (%)		Small single spots (1-2 cells)^e^	Large single spots (> 2 cells)^e^	Twin spots	Total spots		Observed	Control corrected		
*MH*^*3*^											
0	0	40	0.85 (34)	0.00 (00)	0.01 (04)	0.95 (38)	38	1.95			
0	10	38	0.97 (37)	0.08 (03)	0.13 (05)	1.18 (45)	45	2.43	0.48		
0	20	40	0.85 (34)	0.08 (03)	0.00 (00)	0.93 (37)	37	1.90	-0.05		
0	40	32	0.63 (20)	0.03 (01)	0.06 (02)	0.72 (23)	23	1.47	-0.47		
0.125	0	40	2.10 (84)+	1.55 (62)+	2.58(103)+	6.23 (249)+	241	12.35	10.40	87.50	
0.125	10	40	3.35 (134)*	3.20 (128)*	4.65 (186)*	11.20 (448)*	433	22.17	20.24	87.00	94.61
0.125	20	38	3.45 (131)*	3.26 (124)*	3.84 (146)*	10.55(401)*	376	20.28	18.33	90.30	76.25
0.125	40	35	2.71 (95)	1.77 (62)	3.06 (107)	7.54 (264)*	256	14.99	12.87	90.00	23.75
*BH*^*3*^											
0	0	40	0.20 (08)	0.00 (00)	^f^	0.20 (08)	8	0.41			
0.125	0	41	0.68 (28)+	0.10 (04)		0.78 (32)+	32	1.60	1.19		
0.125	10	40	1.20 (48)*	0.28 (11)		1.48 (59)*	59	3.02	2.61		119.33
0.125	20	40	1.00 (40)	0.03 (01)		1.03 (41)	41	2.10	1.69		
0.125	40	31	0.74 (23)	0.03 (01)		0.77 (24)	24	1.59	1.06		

MH flies (*mwh/flr*^*3*^) and BH flies (*mwh/TM3*) were evaluated. ^a^Statistical diagnoses according to Frei and Würgler (1995). *U*-test, two-sided, probability levels: +, p ≤ 0.05 *vs.* untreated control; *, p ≤ 0.05 *vs.* DXR only. ^b^Considering *mwh* clones from *mwh* single and twin spots. ^c^Frequency of clone formation: clones/flies/48,800 cells (without size correction). ^d^Calculated as [DXR alone - (DXR + *Ti*) / DXR alone] X 100, according to Abraham (1994). ^e^Including rare *flr*^*3*^ single spots. ^f^Only mwh single spot can be observed in BH individuals.
